# Analysis of Evolutionary Processes of Species Jump in Waterfowl Parvovirus

**DOI:** 10.3389/fmicb.2017.00421

**Published:** 2017-03-14

**Authors:** Wentao Fan, Zhaoyu Sun, Tongtong Shen, Danning Xu, Kehe Huang, Jiyong Zhou, Suquan Song, Liping Yan

**Affiliations:** ^1^College of Veterinary Medicine, Nanjing Agricultural UniversityNanjing, China; ^2^Jiangsu Engineering Laboratory of Animal Immunology, Institute of Immunology and College of Veterinary Medicine, Nanjing Agricultural UniversityNanjing, China; ^3^Waterfowl Healthy Breeding Engineering Research Center, Guangdong Higher Education InstitutesGuangzhou, China

**Keywords:** species jump, waterfowl parvovirus, phylogeny, epidemiology, evolution, Bayesian inference

## Abstract

Waterfowl parvoviruses are classified into goose parvovirus (GPV) and Muscovy duck parvovirus (MDPV) according to their antigenic features and host preferences. A novel duck parvovirus (NDPV), identified as a new variant of GPV, is currently infecting ducks, thus causing considerable economic loss. This study analyzed the molecular evolution and population dynamics of the emerging parvovirus capsid gene to investigate the evolutionary processes concerning the host shift of NDPV. Two important amino acids changes (Asn-489 and Asn-650) were identified in NDPV, which may be responsible for host shift of NDPV. Phylogenetic analysis indicated that the currently circulating NDPV originated from the GPV lineage. The Bayesian Markov chain Monte Carlo tree indicated that the NDPV diverged from GPV approximately 20 years ago. Evolutionary rate analyses demonstrated that GPV evolved with 7.674 × 10^-4^ substitutions/site/year, and the data for MDPV was 5.237 × 10^-4^ substitutions/site/year, whereas the substitution rate in NDPV branch was 2.25 × 10^-3^ substitutions/site/year. Meanwhile, viral population dynamics analysis revealed that the GPV major clade, including NDPV, grew exponentially at a rate of 1.717 year^-1^. Selection pressure analysis showed that most sites are subject to strong purifying selection and no positively selected sites were found in NDPV. The unique immune-epitopes in waterfowl parvovirus were also estimated, which may be helpful for the prediction of antibody binding sites against NDPV in ducks.

## Introduction

Waterfowl parvovirus infections of geese and ducks have been reported in China, France, Hungary, Germany, Israel, and the USA. GPV causes Derzsy’s disease (gosling plague), which is characterized by high mortality, watery diarrhea, lethargy, anorexia, prostration, and weight loss in young Muscovy ducklings and goslings. MDPV only affects young Muscovy ducklings (2–4 weeks old) and cause 3-W disease, which is characterized with ascites, enteritis, myocarditis, hepatitis, high mortality, and morbidity (Glávits et al., 2005).

Normally, Cherry Valley ducks and mule ducks are resistant to classical goose parvovirus infection (Chen et al., 2016). However, in recent years, a distinct GPV-related parvovirus was successively isolated in mule duck and Cherry Valley duck around the world. The causative agent could proliferate in duck embryos and duck embryo fibroblast cells, but cannot cause a cytopathic effect in primary goose embryo fibroblast cell. The infected ducks show typical symptoms of short beak and dwarfism, with high morbidity and low mortality rates; therefore, the disease was named as beak atrophy and dwarfism syndrome (BADS) (Palya et al., 2009; Li et al., 2016). Since its occurrence in France in 1971, BADS has spread to many European and Asian breeding areas in mule duck, Muscovy duck, and Cherry Valley ducks (Palya et al., 2009). Our surveys show that the recent outbreak of BADS in China has caused severe infection in mule duck and Cherry Valley duck flocks, resulting in immense economic losses (Chen et al., 2015, 2016). Although the GPV-related parvovirus has been isolated, the evolutionary processes of its initial infection and subsequent spread in the new host remain poorly understood.

Several studies have proven that entering and replicating in new host cells are two key factors for a pathogen to infect a novel host (Li et al., 2014; Longdon et al., 2014). Host shift of a pathogen could be monitored by surveillance of potential donor species, observation of specific mutations of a pathogen, and the pathogen’s basic reproductive number (*R*_0_) (Longdon et al., 2015). Moreover, analyses of evolution rate and selection pressure are also necessary for monitoring host shift of a pathogen (Streicker et al., 2010; Mollentze et al., 2014).

According to the International Committee on Taxonomy of Viruses (ICTV) rules, waterfowl parvovirus is classified as a member of *Parvoviridae* family, *Parvovirinae* subfamily, and *Dependoparvovirus* genus. GPV and MDPV all belong to the *Anseriform dependoparvovirus* species. Parvoviruses, characterized with single-stranded DNA genomes, are widely spread from invertebrates to mammal and show a remarkable evolutionary capacity that is different from other DNA viruses (Lópezbueno et al., 2006). The waterfowl parvovirus genome contains two open reading frames (ORFs). The non-structural proteins (NS1 and NS2) are encoded by the left ORF, whereas the right ORF encodes three capsid proteins (VP1, VP2, and VP3). VP2 and VP3 are contained in the carboxyl-terminal portion of VP1 (Istvan et al., 2014). Capsid proteins are important determinants for parvovirus tropism, pathogenicity, and host range (Salganik et al., 2014; Tu et al., 2015). For example, residues on the threefold spike in the capsid proteins determine the host range differences between canine parvovirus (CPV) and feline parvovirus (FPV) (Hueffer and Parrish, 2003). During endosomal trafficking, the minute virus of mice (MVM) virions will expose the N-terminal of VP1 (Mani et al., 2006). Meanwhile, a recombinant capsid VP1 N-terminal with a unique region (VP1u) of human parvovirus B19 became readily recognizable by a specific monoclonal antibody (MAb), whereas the natural capsid was not recognized (Quattrocchi et al., 2012). Therefore, research on VP gene evolutionary processes provided us an opportunity to study the waterfowl parvovirus host range and pathogenicity.

## Materials and Methods

### Waterfowl Parvovirus VP Gene Sequence and Alignment

In this study, a total of 28 waterfowl parvovirus complete capsid genome sequences were obtained from the NCBI GenBank^1^. The alignments were performed using CLUSTAL W (Larkin et al., 2007).

### Structural Modeling of Viral VP Protein

SWISS MODEL (Schwede et al., 2003) was used to build the tertiary structure model of waterfowl VP protein. The waterfowl parvovirus and adeno-associated virus 2 (AAV-2) have high sequence similarity; thus, the AAV-2 capsid structure 5IPI^2^ (Drouin et al., 2016) was chosen as template to create the structural model of waterfowl parvovirus VP protein. The GPV (EU583389) and NDPV (KT343253) sequences were used for the comparative analysis of viral structure. The viral surface was generated using Pymol v1.8 (Delano, 2002).

### Genetics and Evolution Analysis

Phylogenetic analysis was performed using two different methods. Maximum likelihood (ML) analysis was performed using MEGA7.0 with 1000 bootstrap replicates to build the ML tree (Kumar et al., 2016). The evolutionary rates, population growth, and model parameters were all estimated by Bayesian Markov chain Monte Carlo (MCMC) method (Li and Drummond, 2012) implemented in BEAST v2.4.2 package (Drummond and Rambaut, 2007). The JModelTest v2.1.7 (Posada, 2009) was used to choose the suitable evolutionary model that will correspond with the data. The HKY model of nucleotide substitution with a gamma distribution of rate variation including four categories and estimated base frequencies was used to analyze waterfowl parvovirus. In addition, the sequences were partitioned into codon positions 1+2 and 3, with unlinked substitution rates and base frequencies across codon positions. Previous studies indicated that HKY models with codon partitions fit well with most protein coding data sets (Shapiro and Rambaut, 2006; Wu et al., 2013).

Statistical support for specific clade was acquired by calculating the posterior probability of each monophyletic clade. As coalescent priors, two parametric demographic models of population growth (exponential growth and constant size) and a Bayesian skyline plot (BSP, a non-parametric piecewise-constant model) under both strict and relaxed (uncorrelated lognormal) clock conditions were compared (Drummond et al., 2012).

The MCMC chains were run for at least 10 million generations and sampled every 1000 steps. Convergence was estimated on the basis of the effective sampling size (ESS) after a 10% burn-in with Tracer software version 1.6 (Bouckaert et al., 2014). ESSs of >200 were accepted. Uncertainty in the estimates was indicated by 95% highest posterior density (95%HPD) intervals, and the best-fitting models were selected by a Bayes factor (BF, using marginal likelihoods) implemented in BEAST.

As suggested by Kass and Raftery (1995), the strength of the evidence against *H*_0_ is defined as follows: 2ln BF < 2 = none; 2–6 = weak; 6–10 = strong; and > 10 = very strong evidence. A negative 2ln BF means evidence in favor of *H*_0_. Values of >6 were considered significant (Kass and Raftery, 1995). The Tree Annotator program in the BEAST package trees was used to summarize the trees in a target tree by choosing the tree with the maximum posterior probabilities after a 10% burn-in. The final trees were manipulated in the FigTree version 1.3 (Bouckaert et al., 2014) for display purposes. *R*_0_ was calculated from exponential growth rate (r) by the equation *R*_0_ = rD+1, where *D* is the average duration of infectiousness (Pybus et al., 2001). The *D* used for *R*_0_ calculation in this analysis is 0.3–1 month. The choice was based on two things: in most waterfowl parvovirus outbreak cases, the virus usually affects 10-day-old geese and ducks; the growth cycle of cherry valley ducks is about 1 month. Then, the doubling time of the epidemic was calculated by the relation λ = ln(2)/r (Walker et al., 2005).

### Estimation of Positively and Negatively Selected Sites and Selection Pressure

To estimate comprehensively the selection pressure on VP gene of GPV, NDPV, and MDPV, SLAC, FEL, IFEL, and MEME methods were used to calculate non-synonymous (dN) and synonymous (dS) substitution rates at every codon through Datamonkey (Delport et al., 2010). FEL and IFEL methods consider both synonymous and non-synonymous rate variations and may be efficiently parallelized. However, SLAC and FEL methods detect sites under selection at external branches of the phylogenetic tree. By contrast, IFEL method investigates sites along the internal branches (Kosakovsky Pond and Frost, 2005). MEME can consider episodic selective pressure (Murrell et al., 2012). We used four different methods for accurate calculations. The site would be accepted as a negatively or positively selected site if it was simultaneously predicted by two methods. A *P*-value of < 0.05 was used to determine positively (dN > dS) and negatively (dN < dS) selected sites. Genetic Algorithm Recombination Detection (GARD) method (Kosakovsky Pond et al., 2006) has been used for recombination detection through Datamonkey and the dataset included non-recombinant sequences.

### Immune-Epitope Prediction

Immunomedicine Group using Kolaskar and Tongaonkar (1990) method and Protein in DNAStar software based on Jameson–Wolf method (Wolf et al., 1988) were used to predict the B-cell linear epitopes of the standard waterfowl reference strains (GPV: B strain; NDPV: sdlc01 strain; MDPV: FM strain). Epitopes of waterfowl parvovirus VP protein were further identified using Pymol v1.8.

## Results

### Information of Aligned Sequence

The year, country, host, pathogenicity, clinical signs, and accession number of waterfowl parvovirus isolates included in this study are shown in Supplementary Table S1.

### Viral Structure

**FIGURE 1:**
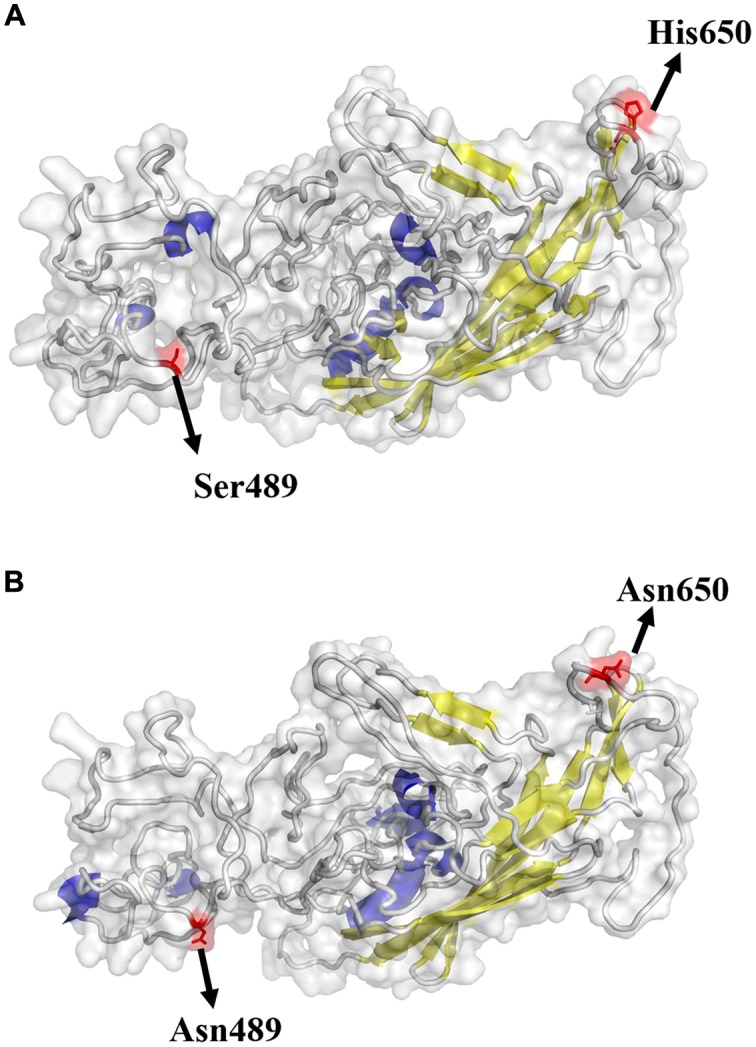
**Structural locations of mutations found in GPV (A) and NDPV (B)**. The capsid structure of an adeno-associated virus (5IPI) was used and aligned to VP of waterfowl parvovirus. The viral surface domain models were generated using Pymol v1.8. Yellow: β-sheet; Blue: α-helix; Gray: loop. The highlighted sites are considered important in host switch.

The possible receptor binding sites of GPV and NDPV were predicted based on the structure of AAV-2. The result demonstrated that the VP protein of NDPV had two important amino acid changes (Asn-489 and Asn-650) when compared with GPV (**Figure 1**). The changed residues were highly conserved in MDPV and NDPV but variable in GPV. Strain-specific mutations were not included in our analysis. The Ser at position 489 changed into Asn in NDPV, and the position may be located in the vicinity of the putative receptor binding site. This change exposed the amino acid at the surface of the NDPV capsid protein. Although position 650 was not located near the putative receptor binding site, the change of His into Asn also led to a projection in viral surface, which may be responsible for host shift for NDPV.

### Genetics and Evolution Analysis of Waterfowl Parvovirus

#### Phylogenetic Analysis

**FIGURE 2:**
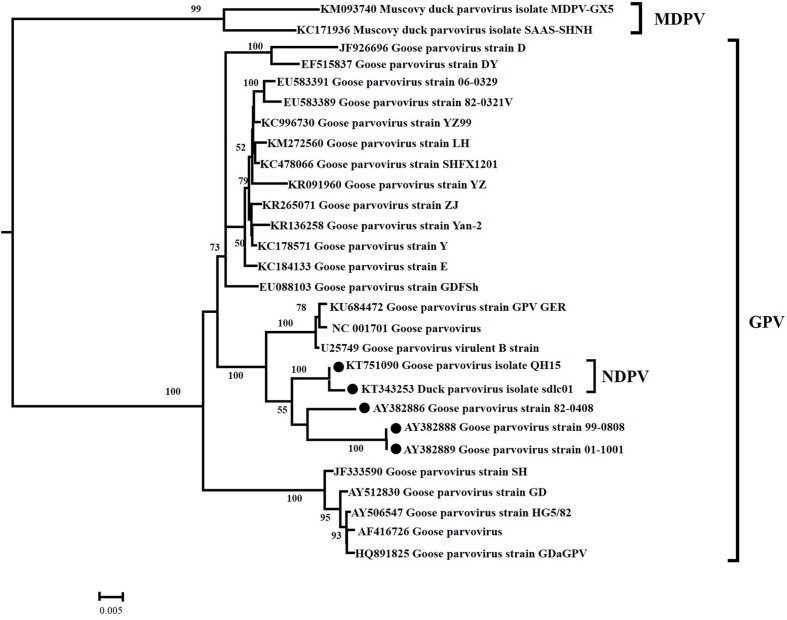
**Maximum likelihood phylogenetic tree of 28 VP gene sequences from waterfowl parvoviruses.** Bootstrap values >50% are shown for relevant nodes. The scale bar represents the number of nucleotide substitutions per site. The name of each isolate is followed by its GenBank number. The 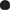 represents NDPV strain and attenuated GPV strain.

In the ML analysis based on capsid gene sequences, waterfowl parvovirus was classified into two classical genetic lineages, GPV and MDPV (**Figure 2**). The ML tree supported the origins of the currently circulating NDPV from GPV lineage. Furthermore, GPV showed high genetic diversity and has evolved into many groups. The important NDPV cases (KT751090 and KT343253) and attenuated GPV strains (AY382888, AY382889, and AY382886) formed individual lineages different from classical GPV strains. The same origins of NDPV and attenuated GPV with high statistical support (Bootstrap values of 100) strongly suggest that NDPV and attenuated GPV had close genetic relationship.

#### Bayesian Evolutionary Analysis

##### Evolutionary rates and tMRCA of waterfowl parvovirus

**FIGURE 3:**
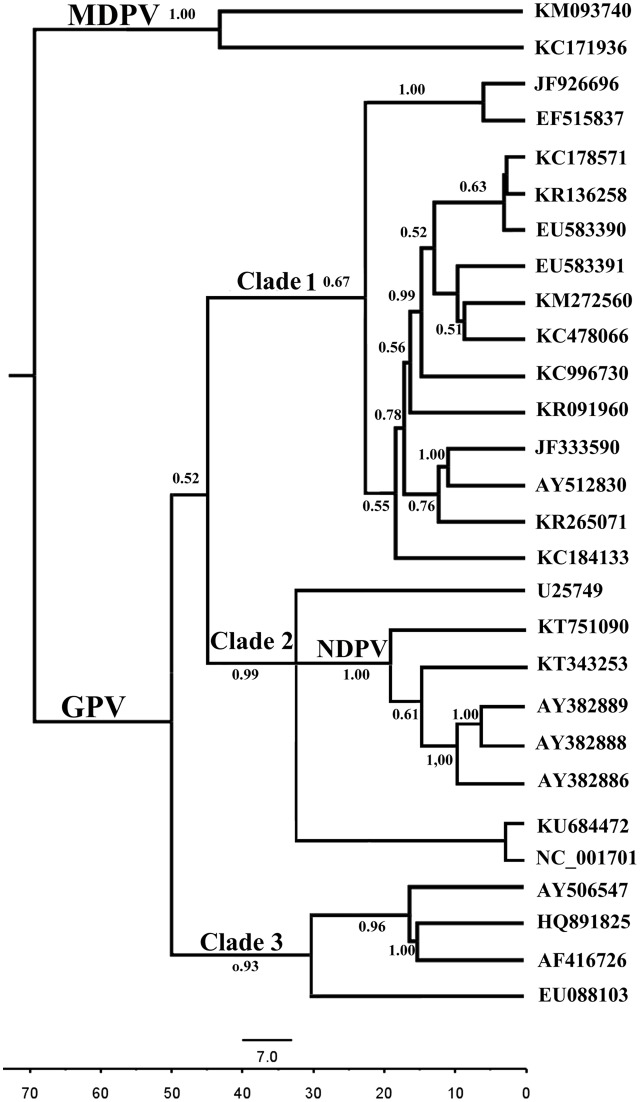
**Bayesian Markov chain Monte Carlo tree of the waterfowl parvovirus based on VP.** The tree was constructed with 10% burn-in by Tree Annotator implemented in the BEAST software package. Posterior probabilities >0.50 are shown at the nodes of each clade. The scale bar represents the unit of time (years).

Both relaxed and strict molecular clock models were performed with three different demographic models (exponential growth, constant population size and a non-parametric BSP). The BFs indicated that the relaxed clock did not fit the data better than the strict clock (Supplementary Table S2). These models show that GPV originated from a common ancestor with MDPV around 70 years ago with a rapid evolution rate of 7.674 × 10^-4^ substitutions/site/year (95% HPD: 6.0946 × 10^-4^, 1.2071 × 10^-3^). The mean rate for two MDPV strains (KM093740 and KC171936) was 5.237 × 10^-4^ substitutions/site/year (95% HPD: 4.32 × 10^-4^, 7.49 × 10^-4^). Hence, despite infecting different host species, these two waterfowl parvovirus evolve at approximately the same rate. Unlike GPV and MDPV, the evolution rate of NDPV is 10 times faster (2.25 × 10^-3^ substitutions/site/year) (Supplementary Table S3), which is similar to a number of RNA viruses, such as HIV-1 (Leitner and Albert, 1999). In another aspect, the GPV strains formed three major clades. The divergence years for these clades were 1971, 1980, and 1986 (**Figure 3**). In addition, the NDPV strain QH15 (KT751090) and the new emerging NDPV stain sdlc01 (KT343253) appeared 20 and 15 years ago, earlier than the time they were isolated. Furthermore, three recent wild attenuated GPV (AY382888, AY382889, and AY382886) were diverged later from NDPV. In fact, these wild GPVs were isolated in 2003, whereas the first BADS outbreak in 1990s was in Taiwan. Therefore, the NDPV may again acquire (have acquired) the ability to infect goose.

##### Population dynamics

After BF comparison of different demographic models using GPV clade 3 strains, we found that the constant size population fitted our data better than the BSP model or exponential growth. On the contrary, BF comparison of GPV clades 1 and 2 isolates displayed strong evidence in favor of exponential growth against constant population size (Supplementary Table S4). The BSP analysis of GPV clades 1 and 2 indicated that GPV infection number remained constant until 2000; thereafter, it underwent exponential growth between the 2000 and 2016 (Supplementary Figure S1). The estimated growth rate was 1.717 year^-1^ (95% HPD: 0.117–2.43); correspondingly, the epidemic doubling time is 0.4 years (credibility limits 0.28–5.8). The average duration of GPV infectiousness ranged from 0.3 to 1 month. Thus, the calculated basic reproductive number (*R*_0_) was between 1.043 and 2.001.

### Selection Pressure Analysis

The dN/dS values, across all VP genes of different lineages, were measured to determine the amount and location of sites under positive selection. The result of site-by-site selection pressure is shown in Supplementary Figure S2. The general dN/dS ratio of 0.1445 and the abundance of negatively selected sites indicated that most sites in VP gene are subjected to purifying selection (Supplementary Table S5). In addition nine sites were estimated as positively selected sites (**Table 1**). The nine sites of specific amino acids were Ser7Lys, Trp19Val, Ala24Val, Gln116His, Gln116Arg, Arg116Thr, Tyr365Leu, Ala366Arg, Asn444Lys, Asn444His, Thr573Ser, Val708Thr, and Val708Ile, respectively (**Table 1**). It is worth noting that the nine positively selected sites are not hypothetic receptor binding sites (Asn-489 and Asn-650), and no positively selected sites was found in NDPV. We also analyzed dN/dS along individual branches of the Neighbor-joining (NJ) tree. Crucially, dN/dS was remarkably elevated on the GPV→MDPV branches of the VP tree. Moreover, two non-synonymous changes (Tyr365Leu and Ala366Arg) occurred in GPV→MDPV branches resulting in dN/dS value of infinity, which is strongly indicative of positive selection. These analyses suggested the selection events might have occurred in evolutionary history. The positive selection may be associated with the host range.

**Table 1:** Positively selected sites of waterfowl parvovirus VP gene.

Sites	7	19	24	116	365	366	444	573	708
AA change	Ser→Lys	Trp→Val	Ala→Val	Gln→His	Tyr→Leu	Ala→Arg	Asn→Lys	Thr→Ser	Val→Thr
				Gln→Arg			Asn→His		Val→Ile
				Arg→Thr					


*AA change: Amino acid changes; dN/dS, non-synonymous rate/synonymous rate; CI, confidence interval. Cut-off *p*-value < 0.05. Mean dN/dS rate (99%CI) = 0.1445 (0.1228–0.165).*

### Predicted Immune-Epitopes in Reference Strains

**FIGURE 4:**
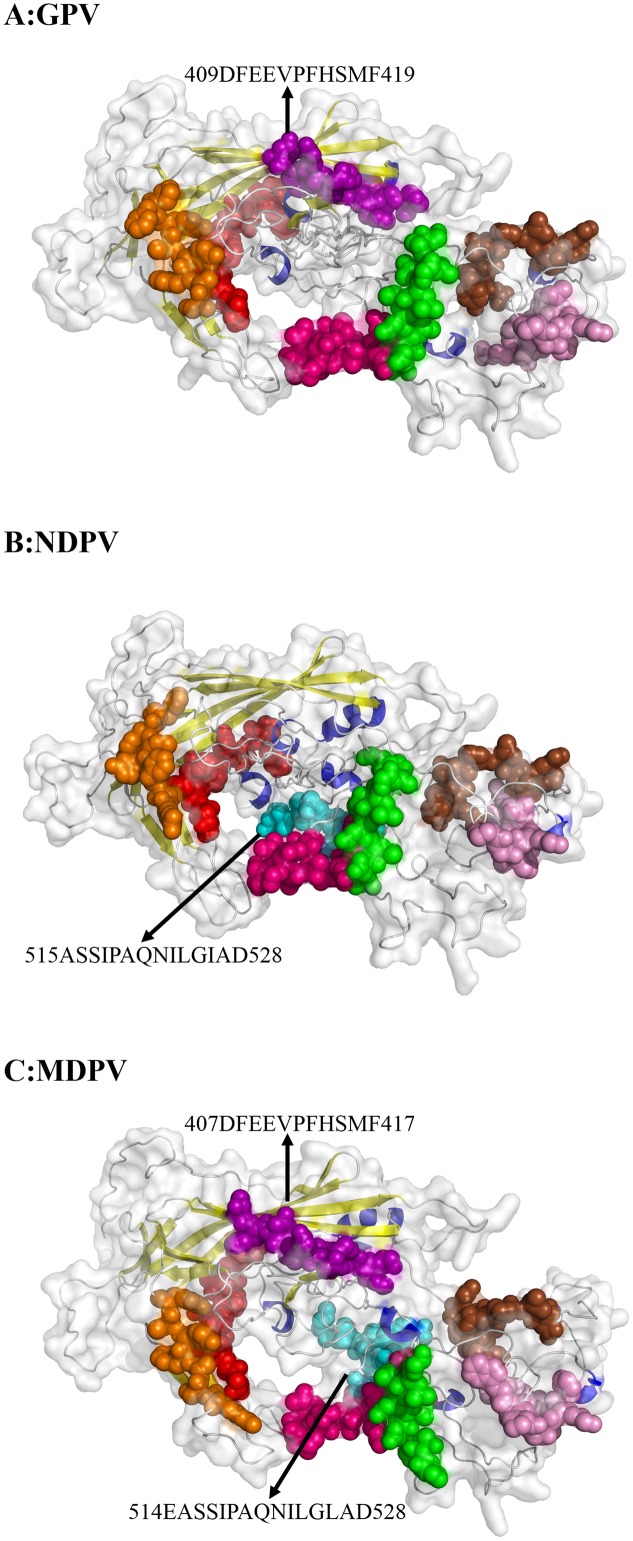
**Predicted immune-epitopes of the reference strains for GPV (A), NDPV (B), and MDPV (C)**. The different predicted epitopes in VP protein are indicated by arrows. Common immune-epitopes are indicated by the same color.

With different software including Immunomedicine Group, Swiss model, and DNAStar, the B-cell linear epitopes in the deduced waterfowl parvovirus amino acid sequences in the reference strains were predicted. Using physicochemical properties of amino acids and experimental antigenic determinant data, Kolaskar and Tongaonkar (1990) derived a parameter to predict antigenic determinants, which works with approximately 75% accuracy. The detailed data are shown in Supplementary Table S6. Most epitopes were similar to the capsid protein of GPV, NDPV, and MDPV. Compared with GPV, NDPV lacked the aa409–aa419 epitope (purple in **Figure 4**), but the aa515–aa528 immune-epitope (blue in **Figure 4**) was only predicted in NDPV. However, the two immune-epitopes (aa409–aa419 for GPV and aa515–aa528 for NDPV) are both included in MDPV genome (aa407–aa417 and aa514–aa528) (**Figure 4**). In addition, positively selected sites (Tyr365Leu and Ala366Arg) are located within the predicted immune-epitopes (aa360–aa374) in MDPV. These immune-epitopes in NDPV and MDPV may be an important index for the prediction of antibody binding sites.

## Discussion

Human, animals, and plants are under constant threat from the emerging viral infectious diseases. The key processes of emerging new viruses are infection and transmission in new hosts (Delaney et al., 2012). To understand these processes, we need to consider the biological processes occurring around the emerging disease. For example, the new host must be exposed to the pathogen, and then the pathogen must be able to infect the new host; finally, the pathogen will keep spreading in the new host population (Longdon et al., 2011).

Parvovirus infections need successful entries into the host cells and many kinds of glycolipids, glycans and glycoproteins might function as receptors. The choice of receptor would determine animal specificity and tissue tropism; and it might influence the endosomal trafficking in host cells (Harbison et al., 2008). Therefore, phylogenetically conserved cell receptors is most important for the virus to enter the cell (Woolhouse, 2002). Previous studies showed that the VP protein of GPV and MDPV has high sequence similarity (70.2 to 70.3%) with AAV-2, in which five amino acids in the VP protein (Arg-484, Arg-487, Lys-532, Arg-585, and Arg588) are directly involved for its receptor binding (Opie et al., 2003; Wu et al., 2006). Shien et al. (2008) suggested that changes in sequence near the receptor binding sites (Arg-487, Lys-571, and Lys/Arg-575) of VP1 might be responsible for attenuation of GPV by using AAV-2 as the model system. As parvoviruses are non-enveloped viruses, the indentations or projections in the capsid surface are responsible for receptors recognition (Smith and Helenius, 2004). In our research, the VP protein of NDPV had two important amino acid changes (Asn-489 and Asn-650) when compared with GPV. The changed amino acid at position 489 is located in the NDPV putative receptor binding site of VP protein. Although the amino acid at position 650 is not near the receptor binding site, the exposed amino acid may form projections or indentations that bind the receptors of duck cells. These changes might lead to the host jump from goose to duck. Similarly, CPV evolved from a virus that is closely related to FPV. The virus got the capability to infect dogs through the acquisition of mutations in capsid. The mutations changed the interactions between virus capsids and the transferrin receptor type 1 (TfR) on canine cells surface (Stucker et al., 2011). However, further investigations should be conducted to verify this hypothesis, such as directed mutagenesis and study of virus–host-cell interactions.

After crossing the species barrier, the pathogen must maintain itself successfully in the new host for its continuous spread, that is, the pathogen should have a reasonable *R*_0_, which satisfies *R*_0_ > 1 (May et al., 2001). If *R*_0_ < 1, despite that the new host repeatedly acquires the pathogen, the spread of infection in the new host will be limited. Conversely, if *R*_0_ > 1, an epidemic may occur (Woolhouse et al., 2005). Few studies about the growth of waterfowl parvovirus epidemic have been reported. Our demographic analysis showed that GPV clades 1 and 2 grew at a high rate (*r* = 1.717 year^-1^), which might cause an epidemic since the time of their radiation. The BSP analysis predicted the effective population size of GPV which showed a constant value from 1966 to 2000, however, the values has been increasing during the recent 15 years. Furthermore a relationship has been found between the effective population size and epidemics of GPV. Since 2000, many studies about GPV outbreak in Sweden, Britain, and China have been reported (Jansson and Palya, 2007; Irvine et al., 2008; Chen et al., 2016). We estimated the *R*_0_ of GPV clades 1 and 2 of 1.043 (assuming *D* = 0.3 month) and 2.001 (assuming *D* = 1 month). Similar *R*_0_ values for the parvovirus B19 (between 1.1 and 1.8) and human bocavirus (between 1.08 and 1.59) were previously estimated by statistical methods (Zehender et al., 2009). These values are lower than the *R*_0_ estimates of pandemic influenza virus but they are similar to the new influenza A virus that has caused outbreaks worldwide (Ferguson et al., 2005; Fraser et al., 2009). These results indicate that NDPV and GPV have high potential for widespread transmission.

B-cell-recognized immune-epitopes of NDPV may be helpful for predicting the antibody binding sites. Previous studies suggested that binding sites in VP domains of parvovirus are related to infections of host cells (Yu et al., 2012). For example, different amino acids in VP2 of CPV compared with amino acids of FPV and mink enteritis virus (MEV) were found (Langeveld et al., 1993). Of these amino acids, aa93–aa103 were found to be critical in immune-epitope recognition for a CPV specific and neutralizing MAb (Parrish et al., 1988). Residues Ala-300 and Thr-301 are critical amino acids for neutralizing MAb specific for both CPV and FPLV (Parrish and Carmichael, 1986). In the present study, many immune-epitopes were predicted in the VP proteins of waterfowl parvovirus. Among them, an immune-epitope of NDPV (aa515–aa528) was estimated as the binding site of VP3 domain, which may lead to the generation of a neutralizing antibody. This prediction provides important information for the development of BADS vaccine.

Most cross-species viral transfers lead to “dead-end” infections in their new hosts without subsequent transmission (Shackelton et al., 2005). The lack of subsequent transmission implies that the transfer of a virus variant that has the capability to replicate in the new host is most important in disease emergence. The association of species jump and subsequent transmission of NDPV with preexisting genetic variation or post-transmission adaptation remains unclear. Phylogenetic analysis showed that NDPV cases and recent wild attenuated GPV strains had close genetic relationship and NDPVs were diverged 20–15 years ago, indicating that NDPV may have been present in some localities for years before it was associated with BADS in China in 2015. Furthermore, our results indicated that the recent wild attenuated GPV was diverged from NDPV, which suggested that NDPV can infect goose with inconspicuous symptoms. This finding might be the reason for the wide malfunction of vaccine in goose industry in Southeastern China (Shao et al., 2015). Although the study demonstrated that most sites are subject to strong purifying selection for NDPV VP gene along with the fast evolutionary process, nine sites had dN/dS values of >1. Thus, these specific amino acids were under positively selected pressure. Especially, Tyr365Leu and Ala366Arg occurred in GPV→MDPV branches resulting in dN/dS value of infinity, indicative of positive selection in MDPV. In general, a strong antigenicity viral protein may undergo strong selection pressure, leading to many positively selected sites present in the antigenic viral protein (Nielsen, 2005). In our study, positively selected sites (Tyr365Leu, Ala366Arg) are located within the predicted immune-epitopes (aa360–aa374) in MDPV. This result suggested that the immune system of host defense mechanisms may act as a selective pressure to MDPV. Nevertheless, the association of species jump of NDPV to selection pressure requires further study.

## Author Contributions

SS and LY designed the study. WF and DX contributed analytic tools. WF, ZS, and TS analyzed data. SS and WF wrote the paper. JZ and KH revised the manuscript. All authors reviewed the results and approved the final version of the manuscript.

## Conflict of Interest Statement

The authors declare that the research was conducted in the absence of any commercial or financial relationships that could be construed as a potential conflict of interest.

## Footnotes

1 https://www.ncbi.nlm.nih.gov/genbank/

2 http://www.rcsb.org
