# Supplementary material for: Analysis of Evolutionary Processes of Species Jump in Waterfowl Parvovirus
Source: Front Microbiol. 2017 Mar 14;8:421. doi: 10.3389/fmicb.2017.00421 (PMC5349109; doi:10.3389/fmicb.2017.00421)
Supplement: Supplementary file 1 [file Data_Sheet_1.DOCX]

**Supplemental Material**

**Analysis of e****volutionary processes of species jumps in waterfowl parvovirus**

Wentao Fan^1^, Zhaoyu Sun^1^, Tongtong Shen^1^, Danning Xu^3^, Kehe Huang^1^, Jiyong Zhou^1, 2^, Suquan Song^1*^ and Liping Yan^1, 2*^

^1^College of Veterinary Medicine, Nanjing Agricultural University, Nanjing 210095, Jiangsu Province, China

^2^Jiangsu Engineering Laboratory of Animal Immunology, Institute of Immunology and College of Veterinary Medicine, Nanjing Agricultural University, Nanjing 210095, Jiangsu Province, China

^3^Waterfowl Healthy Breeding Engineering Research Center, Guangdong Higher Education Institutes, Guangzhou 510225, China

* Corresponding author: E-mail: suquan.song@njau.edu.cn, yanliping@njau.edu.cn

Phone: +86-25-84395789

Fax:+86-25-84395166

**TableS1.** Waterfowl parvovirus sequences used in this study.

| **Isolate** | **Isolated Year** | **Country** | **Host** | **Pathogenicity** | **Clinical signs** | **Accession number** |
| --- | --- | --- | --- | --- | --- | --- |
| GPV 06-0329 | 2006 | Taiwan | Goose | Pathogenic | Typical Derzsy | EU583391 |
| GPV 82-0321 | 1982 | Taiwan | Goose | Pathogenic | Typical Derzsy | EU583390 |
| GPV YZ99-6 | 1999 | China | Goose | Pathogenic | Typical Derzsy | KC996730 |
| GPV LH | 2012 | China | Goose | Pathogenic | Typical Derzsy | KM272560 |
| GPV SHFX1201 | 2012 | China | Swan | Pathogenic | not available | KC478066 |
| GPV YZ | 2013 | China | duck | Pathogenic | Typical Derzsy | KR091960 |
| GPV ZJ | 2013 | China | Goose | Pathogenic | Typical Derzsy | KR265071 |
| GPV Yan-2 | 2013 | China | Goose | Pathogenic | Typical Derzsy | KR136258 |
| GPV Y | 2013 | China | duck | Pathogenic | Typical Derzsy | KC178571 |
| GPV E | 2012 | China | Goose | Pathogenic | Typical Derzsy | KC184133 |
| GPV SH | 2009 | China | Anser | Pathogenic | Typical Derzsy | JF333590 |
| GPV GD | 2003 | China | Goose | Pathogenic | Typical Derzsy | AY512830 |
| GPV YG | 2001 | China | Goose | Pathogenic | Typical Derzsy | AF416726 |
| GPV HG5/82 | 2003 | China | Goose | Pathogenic | Typical Derzsy | AY506547 |
| GPV Gda | 1978 | China | Goose | Pathogenic | Typical Derzsy | HQ891825 |
| GPV Bc | 1995 | Hungry | Goose | Cloning | — | NC001701 |
| GPV B | 1967 | Hungry | Goose | Pathogenic | Typical Derzsy | U25749 |
| GPV GER | 2016 | Poland | Duck | Pathogenic | Typical Derzsy | KU684472 |
| NDPV sdlc01 | 2015 | China | Duck | Moderately pathogenic | Short beak and dwarf | KT343253 |
| NDPV QH15 | 2015 | China | Duck | Moderately pathogenic | Short beak and dwarf | KT751090 |
| Wild GPV | 2003 | Taiwan | Goose | Pathogenic | not available | AY382886 |
| Wild GPV | 2003 | Taiwan | Goose | Pathogenic | not available | AY382889 |
| Wild GPV | 2003 | Taiwan | Goose | Pathogenic | not available | AY382888 |
| GPV GDFSh | 2007 | China | Goose | Pathogenic | Typical Derzsy | EU088103 |
| GPV DY | 2007 | China | Duck | Pathogenic | Typical Derzsy | EF515837 |
| GPV D | 2003 | China | Duck | Pathogenic | Typical Derzsy | JF926696 |
| MDPV GX5 | 2014 | China | Muscovy duck | Pathogenic | Typical Derzsy | KM093740 |
| MDPV SAAS | 2014 | China | Muscovy duck | Pathogenic | Typical Derzsy | KC171936 |

**Table S2.** Bayes factor comparison of strict and relaxed molecular clocks under different population dynamics for GPV.

| **Population dynamics** | **Model comparison**  **(H_0_ vs H_1_)** | **2ln BF** | **Selected**  **hypothesis** | **Evidence** |
| --- | --- | --- | --- | --- |
| Constant population size | Strict vs. relaxed clock | 1.71 | — | No evidence |
| Exponential growth | Strict vs. relaxed clock | 2.54 | H_1_ | Weak |
| Non-parametric Bayesian skyline plot | Strict vs. relaxed clock | 3.18 | H_1_ | Weak |

H_0_: null; H_1_: alternative model; BF: Bayes factor.

**Table S3.** Evolutionary rates and tMRCAs of VP gene of waterfowl parvovirus under a strict clock model. HPD: highest posterior density; tMRCA: time of the most recent common ancestor

|  | GPV | NDPV | MDPV |
| --- | --- | --- | --- |
| Evolutionary rate×10^−4^ substitutions/site/year (95% HPD) | 7.674(6.09-12.07) | 22.5(12.3-23) | 5.237(4.32-7.49) |
| tMRCA years ago(95% HPD) | 70(75-58) | 20(49-15) | 70(72-54) |

**Table S4.** Bayes factor comparison of three demographic models (constant, exponential and BSP) for the different GPV clades.

| **Datasets** | **Model comparison**  **(H0 vs H1)** | **2ln BF** | **Selected hypothesis** | **Evidence** |
| --- | --- | --- | --- | --- |
| Clade 1 | Con vs. BSP | -11.71 | H0 | Very strong |
|  | Con vs. Expo | 53.8 | H1 | Very strong |
|  | BSP vs. Expo | 25.7 | H1 | Very strong |
| Clade 2 | Con vs. BSP | 32.54 | H1 | Very strong |
|  | Con vs. Expo | 82.3 | H1 | Very strong |
|  | BSP vs. Expo | 22.4 | H1 | Very strong |
| Clade 3 | Con vs. BSP | -8.6 | H0 | Strong |
|  | Con vs. Expo | -7.2 | H0 | Strong |
|  | BSP vs. Expo | 1.3 | - | No evidence |

H_0_: null; H_1_: alternative model; BF: Bayes factor; Con: constant; Expo: exponential; BSP: Bayesian skyline plot.

**Table S5.** Negatively selected sites of waterfowl parvovirus VP gene.

|  | SLAC | FEL | IFEL |
| --- | --- | --- | --- |
| Number of negative sites | 41 | 95 | 85 |

SLAC, single likelihood ancestor counting; FEL, fixed effects likelihood; IFEL, internal fixed effects likelihood. Cut-off p-value < 0.05 for SLAC, FEL and IFEL.

\

**Table S6.**Predicted epitopes of the reference strains for waterfowl parvovirus

| **Number** | **GPV** | | |  | **NDPV** | | |  | **MDPV** | | |
| --- | --- | --- | --- | --- | --- | --- | --- | --- | --- | --- | --- |
|  | **Start Position** | **Sequence** | **End Position** |  | **Start Position** | **Sequence** | **End Position** |  | **Start Position** | **Sequence** | **End Position** |
| 1 | 360 | FPSDVYALPQYGYCT | 374 |  | 360 | FPSDVYALPQYGYCT | 374 |  | 360 | FPSDVLRLPQHGYCT | 374 |
| 2 | 388 | SAFYCYFP | 395 |  | 387 | RAFYCYF | 393 |  | 387 | RAFYCYF | 393 |
| 3 | 409 | DFEEVPFHSMF | 419 |  | 423 | YRLNPLDQY | 431 |  | 407 | DFEEVPFHSMF | 417 |
| 4 | 425 | YRLNPLDQY | 433 |  | 461 | PGPKFLDQRVRA | 472 |  | 423 | YRLNPLDQY | 431 |
| 5 | 463 | PGPKFLDQRVRA | 474 |  | 496 | DRQYLLQPGPVSA | 508 |  | 461 | PGPKFLDQRVRA | 472 |
| 6 | 498 | DRQYLLQPGPVSA | 510 |  | 515 | ASSIPAQNILGIAD | 528 |  | 496 | DRQYLLQPGPVSA | 508 |
| 7 | 580 | SDLDVLGALPG | 590 |  | 578 | SDLDVLGALPG | 588 |  | 514 | EASSIPAQNILGLAD | 528 |
| 8 |  |  |  |  |  |  |  |  | 578 | SDLDVLGALPG | 588 |


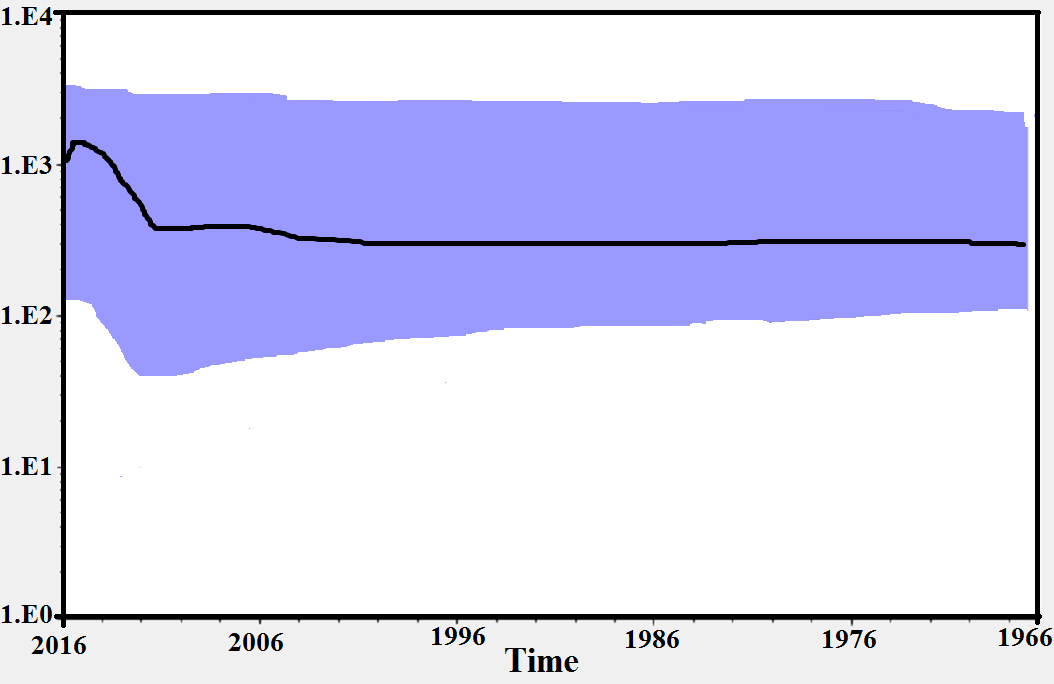


**Figure S1.** Bayesian skyline plot representing the estimates of the effective number of GPV infections (y axis; log10 scale) over time (x axis; calendar years), together with the median estimate (solid line) and credibility interval (filled area).

**
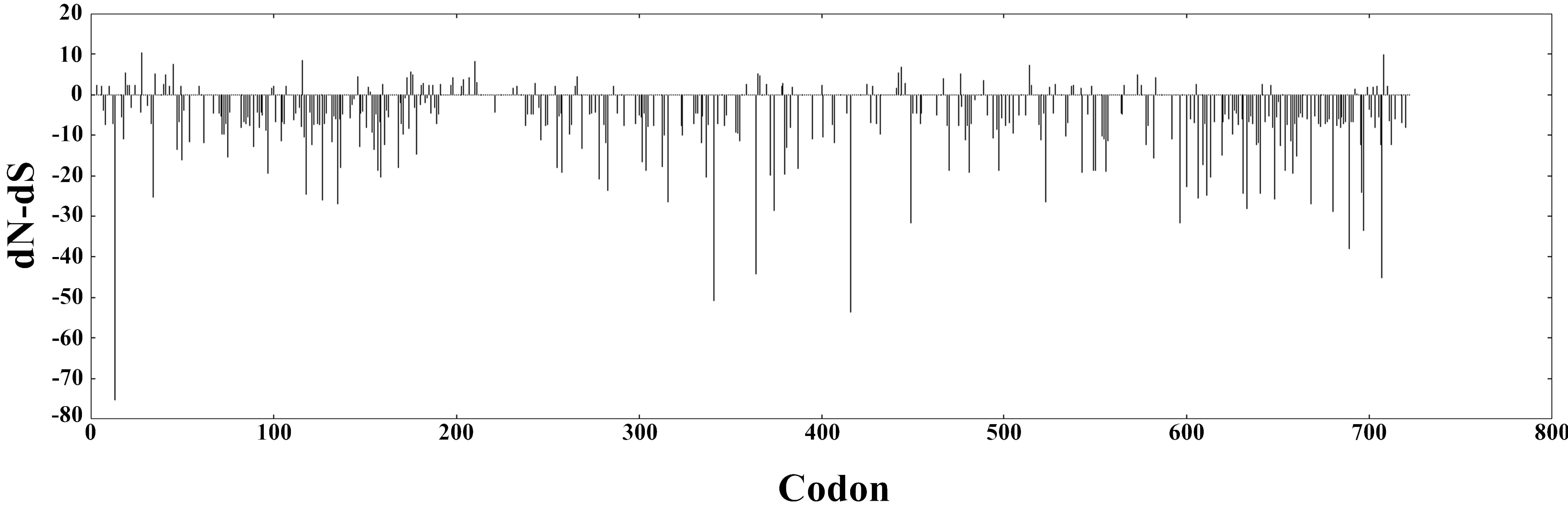
**

**Figure S2.**Site-by-site selection pressure in waterfowl parvovirus VP gene.
